# Rare cutaneous manifestation of zosteriform cutaneous metastases of lung cancer: Two cases and literature review

**DOI:** 10.1002/kjm2.12849

**Published:** 2024-06-06

**Authors:** Ping‐Yi Tsai, Yang Lo

**Affiliations:** ^1^ Department of Dermatology Cathay General Hospital Taipei Taiwan; ^2^ School of Medicine, College of Medicine Fu Jen Catholic University New Taipei Taiwan

Dear Editor,

Cutaneous metastases manifest in 5%–10% of solid tumors,^1^ typically appearing as solitary or widespread papules, and nodules, that are occasionally accompanied by ulceration.^1^ Among these presentations, zosteriform cutaneous metastases are particularly uncommon, particularly in cases of lung cancer.^1^ Herein, we present two cases of zosteriform cutaneous metastases originating from lung cancer.

## CASE 1

**FIGURE 1:**
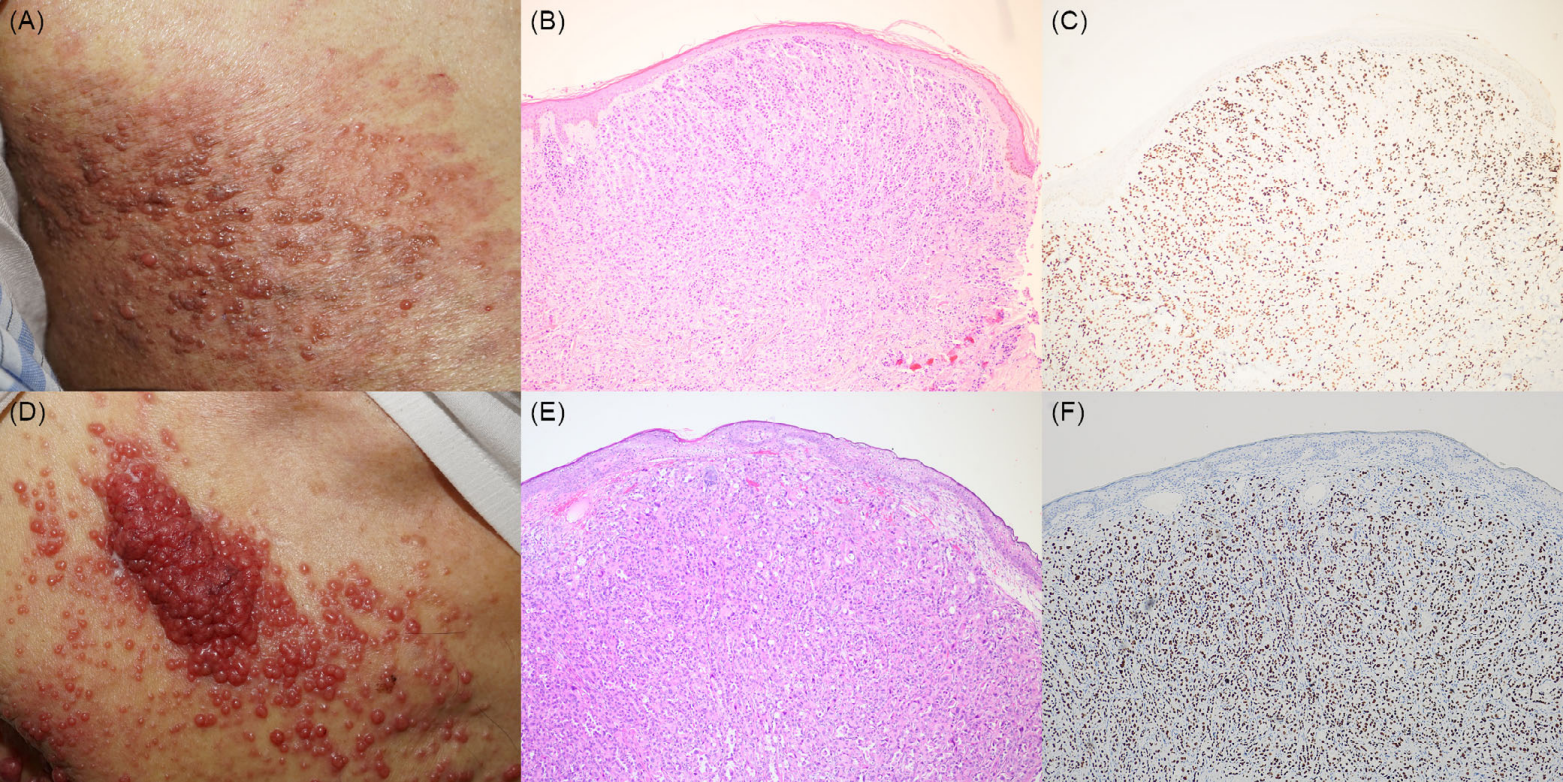
(A) Several erythematous, indurated papules with vesicular appearance and erythematous background on right trunk, consistent with T6 to T8 dermatomes. (B) Histopathologic examination revealed a poorly differentiated carcinoma with sheet like structure, nests and cell cords invading the dermis (original magnification ×100). (C) Immunohistochemical stain showed positive result of thyroid transcription factor 1 (original magnification ×100). (D) Several pink‐reddish shiny pinhead‐sized papules on right anterior trunk, consistent with T1 to T4 dermatomes. (E) Histopathologic examination revealed moderately to poorly differentiated adenocarcinoma with acinar, solid, and micropapillary growth patterns invading the fibrotic dermis (original magnification ×100). (F) Immunohistochemical stain showed positive result of thyroid transcription factor 1 (original magnification ×100).

A 72‐year‐old man with a history of lung adenocarcinoma presented to our clinic with several asymptomatic papules on his right trunk for the past 2 weeks. Upon examination, several erythematous, indurated papules with a vesicular appearance and an erythematous background were noted on the right trunk, corresponding to the T6 to T8 dermatomes (Figure 1A). Histopathologic analysis revealed a poorly differentiated carcinoma displaying sheet‐like structure, nests and cell cords invading the dermis (Figure 1B). Immunohistochemical staining demonstrated a positive result for thyroid transcription factor 1 (Figure 1C). Consequently, a diagnosis of metastatic adenocarcinoma originating from lung cancer with a zosteriform presentation was made.

## CASE 2

A 91‐year‐old man with a history of hepatoma and sick sinus syndrome, with permanent pacemaker implantation, presented to our clinic with an asymptomatic skin lump on the chest for the past 2 months. Skin examination revealed pink‐reddish, vegetative, shiny, and pinhead‐sized papules with confluent plaques on the right anterior chest (Figure 1D). Histopathologic examination revealed moderately to poorly differentiated adenocarcinoma with acinar, solid and micropapillary growth patterns invading the fibrotic dermis (Figure 1E). Immunohistochemical staining revealed positive results for CK7 and thyroid transcription factor 1 (Figure 1F). Consequently, a diagnosis of metastatic adenocarcinoma originating from lung cancer with a zosteriform presentation was established.

## DISCUSSION

Zosteriform cutaneous metastases are a rare presentation, with limited number of reported cases documented in the literature.^1^, ^2^, ^3^, ^4^, ^5^ We reviewed previously reported cases of zosteriform cutaneous metastases originating from lung cancer (Table S1).^1^, ^2^, ^3^, ^4^, ^5^ Among the 13 cases analyzed, the majority had cutaneous metastases on the ipsilateral face and trunk, with the exception of case 6. This observation suggests the mechanism of lymphovascular invasion by tumor cells.^1^, ^2^, ^3^, ^4^, ^5^ Furthermore, 6 of the 13 patients reported experiencing pain, a symptom that could lead to misdiagnosis or delayed diagnosis in the case of a zosteriform presentation.^1^, ^2^, ^3^, ^4^

The exact mechanism underlying zosteriform cutaneous metastases remains unknown, although several theories have been proposed. One such theory suggests, a Koebner or Koebner like phenomenon, where in skin previously affected by the varicella zoster virus may become more susceptible to the development of leukemic infiltrates at the same site.^1^, ^2^ However, this explanation seems less plausible for our cases, as neither patient had a history of varicella or herpes zoster infection at the site of cutaneous metastases. Additionally, in our review, the majority of reported patients did not have a history of herpes zoster.^1^, ^2^, ^3^, ^4^, ^5^ Another proposed mechanism involves retrograde migration through the lymphatics or blood vessels.^1^, ^2^, ^3^, ^5^ Maki et al. reported a case of zosteriform cutaneous metastasis of squamous cell lung carcinoma originating from the right upper lobe. Autopsy findings revealed bilateral hilar lymph node metastasis, with tumor cells present in lymphatic vessels near the subclavian veins.^5^ These findings suggest that retrograde migration through lymphatic vessels might be a more plausible mechanism in our cases. Furthermore, although previous studies have indicated poor prognosis for cutaneous metastasis from lung cancer, no report has suggested poorer prognosis with zosteriform cutaneous metastases.

In conclusion, these two cases underscore the importance of considering cutaneous metastases when encountering zosteriform presentation. Healthcare providers should remain vigilant for zosteriform metastases in patients with a clinical history of internal malignancy, particularly in case of an inadequate reaction to anti‐herpes treatment.

## CONFLICT OF INTEREST STATEMENT

All authors declare no conflict of interest.

## Supporting information


**Table S1.** Fifteenth cases of cutaneous zosteriform metastases of lung cancer.
