# Supplementary material for: Rare cutaneous manifestation of zosteriform cutaneous metastases of lung cancer: Two cases and literature review
Source: Kaohsiung J Med Sci. 2024 Jun 6;40(8):768–9. doi: 10.1002/kjm2.12849 (PMC11895620; doi:10.1002/kjm2.12849)
Supplement: Supplementary file 1 — Table S1. Fifteenth cases of cutaneous zosteriform metastases of lung cancer. [file KJM2-40-768-s001.docx]

| Author | Year reported | Age/sex | Primary tumor | Site of cutaneous lesions | Time from first  diagnosis to  zosteriform  metastases | Pain |
| --- | --- | --- | --- | --- | --- | --- |
| Hodge et al. | 1979 | 57/M | Adenocarcinoma of lung | Ipsilateral T5-T7 | 3 weeks | NM* |
| Matarasso et al.^6^ | 1986 | 65/M | Adenocarcinoma of lung (LUL†) | Ipsilateral T7(left) | 5 months | + |
| Bianchi et al.^7^ | 2000 | 71/M | Bronchogenic carcinoma (left primary bronchus) | Lower face | simultaneous | + |
| Kikuchi et al.^8^ | 2001 | 69/M | Lung cancer | Ipsilateral T8-T9(right) | 1 month | + |
| LeSueur et al.^2^ | 2004 | 66/F | Bronchogenic adenocarcinoma | Right thigh | 1 year | + |
| Subramanyam et al.^9^ | 2016 | 75/M | Adenocarcinoma of lung (RUL‡) | Left C4 | simultaneous | - |
| Aiempanakit et al.^10^ | 2017 | 68/M | Adenocarcinoma of lung | Left T4 | 1 year | - |
| Wark et al.^11^ | 2019 | 78/M | Adenocarcinoma of lung (RUL) | Right C4-C5 | simultaneous | - |
| Wang et al.^3^ | 2020 | 49/F | Adenocarcinoma of lung | Left breast(T4) | 1 year | + |
| Dagdele et al.^4^ | 2020 | 52/M | Lung cancer(R) | Right T5-T7 | 17 years | - |
| Dagdele et al.^4^ | 2020 | 62/F | Lung cancer(L) | Left T3-T5 | 4 months | + |
| Dagdele et al.^4^ | 2020 | 52/M | Lung cancer(R) | Right T4-T6 | 51 months | - |
| Maki et al.^5^ | 2021 | 68/M | Squamous  cell lung carcinoma (RUL) | Right T2-T4 | 4 months | - |
| Present study | 2024 | 72/M | Adenocarcinoma of lung (RML§) | Right T6-T8 | 47 months | - |
| Present study | 2024 | 91/M | Adenocarcinoma of lung | Right T1-T4 | simultaneous | - |

Table 1

Table legend:

**Table 1** Fifteenth cases of cutaneous zosteriform metastases of lung cancer.

*NM: not mentioned; †LUL: left upper lobe; ‡RUL: right upper lobe; §RML: right middle lobe

Reference:

2. LeSueur BW, Abraham RJ, DiCaudo DJ, O'Connor WJ. Zosteriform skin metastases. Int J Dermatol. 2004;43(2):126–128.

1. Wang Y, Xue R. Cutaneous metastases from lung adenocarcinoma. Case Rep Dermatol Med. 2020;12(2020):8880604.
2. Dagdelen D, Karadag AS, Akdeniz N, Ozkanli SS, Gurel MS. Zosteriform cutaneous metastasis: a case series. Dermatol Ther. 2020;33(6):e14137.
3. Maki Y, Kimizuka Y, Murakami K, Sato K, Sasaki H, Yamamoto T, et al. Zosteriform skin metastasis caused by retrograde lymphatic migration of metastatic squamous cell lung carcinoma. BMC Pulm Med. 2021;21(1):41.
4. Matarasso SL, Rosen T. Zosteriform metastasis: case presentation and review of the literature. J Dermatol Surg Oncol. 1988; 14 (7):774-778.
5. Bianchi L, Orlandi A, Carboni I, Costanzo A, Chimenti S. Zosteriform metastasis of occult bronchogenic carcinoma. Acta Derm Venereol. 2000; 80 (5):391-392.
6. Kikuchi Y, Matsuyama A, Nomura K. Zosteriform metastatic skin cancer: report of three cases and review of the literature. Dermatology. 2001; 202 (4):336-338.
7. Subramanyam P, Palaniswamy SS, Tewari A. Zosteriform cutaneous metastases from an occult primary malignancy of lung identified by whole-body FDG PETCT imaging. Indian J Nucl Med. 2016; 31 (4):286-288.
8. Aiempanakit K, Sangmala S, Chiratikarnwong K, Auepemkiate S. Zoster-like cutaneous metastatic adenocarcinoma of the lung: A case report. Respir Med Case Rep. 2017; 22:274-276.
9. Wark KJL, Mahendran M, Tatian A, Singh A, Woods J, Aravindan A. Zosteriform cutaneous metastases: an unusual presentation of metastatic lung carcinoma. Respirol Case Rep. 2020; 8 (2):e00515.
